# {4,4′,6,6′-Tetra­bromo-2,2′-[(2,2-dimethyl­propane-1,3-di­yl)bis­(nitrilo­methanylyl­idene)]diphenolato}copper(II)

**DOI:** 10.1107/S1600536812009397

**Published:** 2012-03-10

**Authors:** Hadi Kargar, Reza Kia, Mahbubeh Haghshenas, Muhammad Nawaz Tahir

**Affiliations:** a Department of Chemistry, Payame Noor University, PO Box 19395-3697 Tehran, I. R. of IRAN; b X-ray Crystallography Laboratory, Plasma Physics Research Center, Science and Research Branch, Islamic Azad University, Tehran, Iran, and, Department of Chemistry, Science and Research Branch, Islamic Azad University, Tehran, Iran; c Department of Physics, University of Sargodha, Punjab, Pakistan

## Abstract

In the title compound, [Cu(C_19_H_16_Br_4_N_2_O_2_)], the Cu^II^ ion and the substituted C atom of the diamine fragment lie on a crystallographic twofold rotation axis. The geometry around the Cu^II^ ion is distorted square-planar, which is defined by the N_2_O_2_ donor atoms of the coordinated Schiff base ligand. The dihedral angle between the symmetry-related substituted benzene rings is 25.33 (14)°. The crystal structure is stabilized by an inter­molecular π–π inter­action [centroid–centroid distance = 3.8891 (18) Å].

## Related literature

For standard bond lengths, see: Allen *et al.* (1987 ▶). For applications of Schiff base ligands in coordination chemistry, see: Granovski *et al.* (1993 ▶); Blower (1998 ▶). For a related structure, see: Kargar *et al.* (2008 ▶).
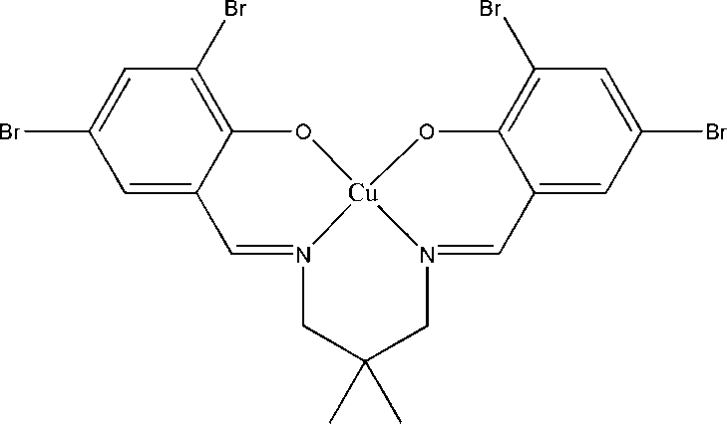


## Experimental

### 

#### Crystal data

[Cu(C_19_H_16_Br_4_N_2_O_2_)]
*M* *_r_* = 687.52
Orthorhombic, 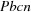

*a* = 16.3594 (8) Å
*b* = 15.5106 (8) Å
*c* = 8.4686 (4) Å
*V* = 2148.86 (18) Å^3^
*Z* = 4
Mo *K*α radiation
μ = 8.47 mm^−1^
*T* = 291 K
0.21 × 0.12 × 0.08 mm

#### Data collection

Bruker SMART APEXII CCD area-detector diffractometer
Absorption correction: multi-scan (*SADABS*; Bruker, 2005 ▶) *T*_min_ = 0.269, *T*_max_ = 0.551
9913 measured reflections
2537 independent reflections
1625 reflections with *I* > 2σ(*I*)
*R*_int_ = 0.052

#### Refinement

*R*[*F* ^2^ > 2σ(*F* ^2^)] = 0.039
*wR*(*F* ^2^) = 0.078
*S* = 1.00
2537 reflections
128 parameters
H-atom parameters constrained
Δρ_max_ = 0.58 e Å^−3^
Δρ_min_ = −0.59 e Å^−3^

### 

Data collection: *APEX2* (Bruker, 2005 ▶); cell refinement: *SAINT* (Bruker, 2005 ▶); data reduction: *SAINT*; program(s) used to solve structure: *SHELXTL* (Sheldrick, 2008 ▶); program(s) used to refine structure: *SHELXTL*; molecular graphics: *SHELXTL*; software used to prepare material for publication: *SHELXTL* and *PLATON* (Spek, 2009 ▶).

## Supplementary Material

Crystal structure: contains datablock(s) global, I. DOI: 10.1107/S1600536812009397/bv2199sup1.cif

Structure factors: contains datablock(s) I. DOI: 10.1107/S1600536812009397/bv2199Isup2.hkl

Additional supplementary materials:  crystallographic information; 3D view; checkCIF report

## Figures and Tables

**Fig. 1.**
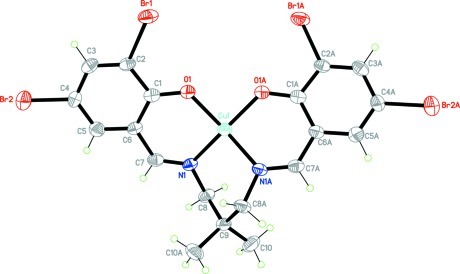
The ORTEP plot of the title compound, showing 40% probability displacement ellipsoids and the atomic numbering.

**Fig. 2.**
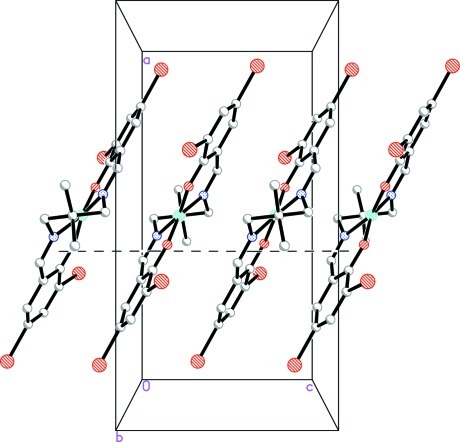
The packing diagram of the title compound viewed down the b-axis showing linking of molecules through the intermolecular π-π intearctions (dashed lines). The hydrogen atoms omitted for clarity.

## supplementary crystallographic information

### Comment

Schiff base complexes are one of the most important stereochemical models in transition metal coordination chemistry, with the ease of preparation and structural variations (Granovski *et al.*, 1993; Blower (1998)).

The asymmetric unit of the title compound, Fig. 1, comprises half of a potentially tetradentate Schiff base ligand. The bond lengths (Allen *et al.*, 1987) and angles are within the normal ranges. The Cu1 and C9 atoms lie on crystallographic two-fold rotation axis. The crystal structure is further stabilized by the intermolecular π-π interaction, (Fig. 2), [*Cg*1···*Cg*1^i^ = 3.8891 (18)Å; (i) 1 - *X*, 1 - *Y*, -*Z*; *Cg*1 is the centroid of Cu(1)/O(1)/C(1)/C(6)/C(7)/N(1) ring].

### Experimental

The title compound was synthesized by adding 3,5-dibromo-salicylaldehyde-2,2-dimethyl-1,3- propanediamine (2 mmol) to a solution of CuCl_2_. 4H_2_O (2.1 mmol) in ethanol (30 ml). The mixture was refluxed with stirring for half an hour. The resultant solution was filtered. Green single crystals of the title compound suitable for *X*-ray structure determination were recrystallized from ethanol by slow evaporation of the solvents at room temperature over several days.

### Refinement

All hydrogen atoms were positioned geometrically with C—H = 0.93-0.97 Å and included in a riding model and treated as riding atoms: C—H = 0.93, 0.96 and 0.97 Å for CH, CH_3_ and CH_2_ H-atoms, respectively, with U_iso_ (H) = k x U_eq_(C), where k = 1.5 for CH_3_ H-atoms, and k = 1.2 for all other H-atoms..

### Crystal data

**Table:**

[Cu(C_19_H_16_Br_4_N_2_O_2_)]	*F*(000) = 1316
*M**_r_* = 687.52	*D*_x_ = 2.125 Mg m^−^^3^
Orthorhombic, *P**b**c**n*	Mo *K*α radiation, λ = 0.71073 Å
Hall symbol: -P 2n 2ab	Cell parameters from 1535 reflections
*a* = 16.3594 (8) Å	θ = 2.5–27.5°
*b* = 15.5106 (8) Å	µ = 8.47 mm^−^^1^
*c* = 8.4686 (4) Å	*T* = 291 K
*V* = 2148.86 (18) Å^3^	Block, green
*Z* = 4	0.21 × 0.12 × 0.08 mm

### Data collection

**Table:**

Bruker SMART APEXII CCD area-detector diffractometer	2537 independent reflections
Radiation source: fine-focus sealed tube	1625 reflections with *I* > 2σ(*I*)
Graphite monochromator	*R*_int_ = 0.052
φ and ω scans	θ_max_ = 28.0°, θ_min_ = 1.8°
Absorption correction: multi-scan (*SADABS*; Bruker, 2005)	*h* = −21→18
*T*_min_ = 0.269, *T*_max_ = 0.551	*k* = −20→13
9913 measured reflections	*l* = −10→7

### Refinement

**Table:**

Refinement on *F*^2^	Primary atom site location: structure-invariant direct methods
Least-squares matrix: full	Secondary atom site location: difference Fourier map
*R*[*F*^2^ > 2σ(*F*^2^)] = 0.039	Hydrogen site location: inferred from neighbouring sites
*wR*(*F*^2^) = 0.078	H-atom parameters constrained
*S* = 1.00	*w* = 1/[σ^2^(*F*_o_^2^) + (0.0202*P*)^2^ + 1.7635*P*] where *P* = (*F*_o_^2^ + 2*F*_c_^2^)/3
2537 reflections	(Δ/σ)_max_ = 0.001
128 parameters	Δρ_max_ = 0.58 e Å^−^^3^
0 restraints	Δρ_min_ = −0.59 e Å^−^^3^

### Special details

**Table:**

Geometry. All esds (except the esd in the dihedral angle between two l.s. planes) are estimated using the full covariance matrix. The cell esds are taken into account individually in the estimation of esds in distances, angles and torsion angles; correlations between esds in cell parameters are only used when they are defined by crystal symmetry. An approximate (isotropic) treatment of cell esds is used for estimating esds involving l.s. planes.
Refinement. Refinement of F^2^ against ALL reflections. The weighted R-factor wR and goodness of fit S are based on F^2^, conventional R-factors R are based on F, with F set to zero for negative F^2^. The threshold expression of F^2^ > 2sigma(F^2^) is used only for calculating R-factors(gt) etc. and is not relevant to the choice of reflections for refinement. R-factors based on F^2^ are statistically about twice as large as those based on F, and R- factors based on ALL data will be even larger.

### Fractional atomic coordinates and isotropic or equivalent isotropic displacement parameters (Å^2^)

**Table:**

	*x*	*y*	*z*	*U*_iso_*/*U*_eq_	
Br1	0.33450 (3)	0.74146 (3)	0.18143 (5)	0.05098 (16)	
Br2	0.10313 (3)	0.54552 (4)	−0.15415 (7)	0.0733 (2)	
Cu1	0.5000	0.48415 (4)	0.2500	0.03410 (18)	
N1	0.4444 (2)	0.39656 (19)	0.1258 (3)	0.0332 (7)	
O1	0.42323 (17)	0.57112 (16)	0.1958 (3)	0.0395 (7)	
C1	0.3547 (2)	0.5618 (2)	0.1192 (4)	0.0341 (9)	
C2	0.3023 (2)	0.6335 (2)	0.0965 (4)	0.0341 (9)	
C3	0.2301 (2)	0.6285 (3)	0.0162 (4)	0.0406 (10)	
H3	0.1982	0.6776	0.0030	0.049*	
C4	0.2042 (2)	0.5504 (3)	−0.0458 (5)	0.0417 (10)	
C5	0.2520 (3)	0.4788 (3)	−0.0299 (4)	0.0425 (10)	
H5	0.2348	0.4267	−0.0730	0.051*	
C6	0.3270 (2)	0.4831 (2)	0.0511 (4)	0.0342 (9)	
C7	0.3757 (3)	0.4058 (2)	0.0534 (4)	0.0368 (10)	
H7	0.3560	0.3585	−0.0022	0.044*	
C8	0.4911 (3)	0.3163 (2)	0.1017 (4)	0.0404 (10)	
H8A	0.5453	0.3310	0.0635	0.048*	
H8B	0.4644	0.2825	0.0204	0.048*	
C9	0.5000	0.2606 (3)	0.2500	0.0390 (14)	
C10	0.5749 (3)	0.2038 (3)	0.2270 (5)	0.0574 (13)	
H10A	0.6231	0.2391	0.2253	0.086*	
H10B	0.5703	0.1733	0.1288	0.086*	
H10C	0.5786	0.1633	0.3123	0.086*	

### Atomic displacement parameters (Å^2^)

**Table:**

	*U*^11^	*U*^22^	*U*^33^	*U*^12^	*U*^13^	*U*^23^
Br1	0.0710 (3)	0.0317 (2)	0.0503 (3)	0.0104 (2)	−0.0024 (2)	−0.0063 (2)
Br2	0.0527 (3)	0.0645 (4)	0.1027 (4)	0.0055 (3)	−0.0303 (3)	0.0001 (3)
Cu1	0.0387 (4)	0.0275 (3)	0.0361 (4)	0.000	−0.0012 (3)	0.000
N1	0.041 (2)	0.0256 (17)	0.0334 (17)	0.0063 (16)	0.0008 (15)	−0.0012 (14)
O1	0.0416 (17)	0.0295 (14)	0.0475 (16)	0.0014 (13)	−0.0063 (13)	−0.0027 (13)
C1	0.042 (3)	0.031 (2)	0.030 (2)	0.002 (2)	0.0088 (18)	0.0010 (18)
C2	0.043 (2)	0.029 (2)	0.031 (2)	0.0058 (19)	0.0072 (18)	−0.0018 (18)
C3	0.044 (3)	0.039 (2)	0.039 (2)	0.013 (2)	0.0078 (19)	0.002 (2)
C4	0.035 (2)	0.046 (3)	0.045 (2)	0.006 (2)	−0.0028 (19)	0.001 (2)
C5	0.045 (3)	0.039 (2)	0.043 (2)	−0.002 (2)	−0.002 (2)	−0.002 (2)
C6	0.041 (2)	0.029 (2)	0.032 (2)	0.005 (2)	0.0022 (17)	0.0001 (18)
C7	0.049 (3)	0.029 (2)	0.032 (2)	0.001 (2)	0.0022 (19)	−0.0042 (18)
C8	0.052 (3)	0.031 (2)	0.038 (2)	0.012 (2)	−0.0001 (19)	−0.0050 (19)
C9	0.047 (4)	0.026 (3)	0.044 (3)	0.000	−0.007 (3)	0.000
C10	0.064 (3)	0.046 (3)	0.062 (3)	0.017 (3)	−0.013 (2)	0.005 (2)

### Geometric parameters (Å, º)

**Table:**

Br1—C2	1.897 (4)	C4—C5	1.364 (6)
Br2—C4	1.893 (4)	C5—C6	1.407 (5)
Cu1—O1^i^	1.899 (3)	C5—H5	0.9300
Cu1—O1	1.899 (3)	C6—C7	1.440 (5)
Cu1—N1^i^	1.944 (3)	C7—H7	0.9300
Cu1—N1	1.944 (3)	C8—C9	1.531 (5)
N1—C7	1.288 (5)	C8—H8A	0.9700
N1—C8	1.475 (5)	C8—H8B	0.9700
O1—C1	1.303 (5)	C9—C10^i^	1.522 (5)
C1—C2	1.418 (5)	C9—C10	1.522 (5)
C1—C6	1.425 (5)	C9—C8^i^	1.531 (5)
C2—C3	1.364 (5)	C10—H10A	0.9600
C3—C4	1.387 (6)	C10—H10B	0.9600
C3—H3	0.9300	C10—H10C	0.9600
			
O1^i^—Cu1—O1	89.50 (16)	C5—C6—C1	121.0 (4)
O1^i^—Cu1—N1^i^	93.23 (12)	C5—C6—C7	116.8 (4)
O1—Cu1—N1^i^	159.45 (11)	C1—C6—C7	122.1 (4)
O1^i^—Cu1—N1	159.45 (11)	N1—C7—C6	125.6 (4)
O1—Cu1—N1	93.23 (12)	N1—C7—H7	117.2
N1^i^—Cu1—N1	91.32 (18)	C6—C7—H7	117.2
C7—N1—C8	118.7 (3)	N1—C8—C9	114.3 (3)
C7—N1—Cu1	126.0 (3)	N1—C8—H8A	108.7
C8—N1—Cu1	115.0 (3)	C9—C8—H8A	108.7
C1—O1—Cu1	127.6 (2)	N1—C8—H8B	108.7
O1—C1—C2	120.1 (4)	C9—C8—H8B	108.7
O1—C1—C6	124.8 (4)	H8A—C8—H8B	107.6
C2—C1—C6	115.1 (4)	C10^i^—C9—C10	109.2 (5)
C3—C2—C1	123.2 (4)	C10^i^—C9—C8^i^	107.3 (2)
C3—C2—Br1	118.7 (3)	C10—C9—C8^i^	110.8 (2)
C1—C2—Br1	118.2 (3)	C10^i^—C9—C8	110.8 (2)
C2—C3—C4	120.2 (4)	C10—C9—C8	107.3 (2)
C2—C3—H3	119.9	C8^i^—C9—C8	111.3 (4)
C4—C3—H3	119.9	C9—C10—H10A	109.5
C5—C4—C3	119.9 (4)	C9—C10—H10B	109.5
C5—C4—Br2	121.1 (3)	H10A—C10—H10B	109.5
C3—C4—Br2	119.0 (3)	C9—C10—H10C	109.5
C4—C5—C6	120.6 (4)	H10A—C10—H10C	109.5
C4—C5—H5	119.7	H10B—C10—H10C	109.5
C6—C5—H5	119.7		
			
O1^i^—Cu1—N1—C7	−102.7 (4)	C2—C3—C4—Br2	179.2 (3)
O1—Cu1—N1—C7	−5.5 (3)	C3—C4—C5—C6	1.0 (6)
N1^i^—Cu1—N1—C7	154.5 (4)	Br2—C4—C5—C6	−179.9 (3)
O1^i^—Cu1—N1—C8	70.7 (4)	C4—C5—C6—C1	0.3 (6)
O1—Cu1—N1—C8	167.9 (2)	C4—C5—C6—C7	−176.2 (4)
N1^i^—Cu1—N1—C8	−32.2 (2)	O1—C1—C6—C5	180.0 (3)
O1^i^—Cu1—O1—C1	167.4 (3)	C2—C1—C6—C5	−0.9 (5)
N1^i^—Cu1—O1—C1	−94.7 (4)	O1—C1—C6—C7	−3.7 (6)
N1—Cu1—O1—C1	7.8 (3)	C2—C1—C6—C7	175.4 (3)
Cu1—O1—C1—C2	176.5 (2)	C8—N1—C7—C6	−173.3 (3)
Cu1—O1—C1—C6	−4.5 (5)	Cu1—N1—C7—C6	−0.1 (6)
O1—C1—C2—C3	179.4 (3)	C5—C6—C7—N1	−177.5 (4)
C6—C1—C2—C3	0.2 (5)	C1—C6—C7—N1	6.1 (6)
O1—C1—C2—Br1	−0.4 (5)	C7—N1—C8—C9	−115.0 (4)
C6—C1—C2—Br1	−179.5 (3)	Cu1—N1—C8—C9	71.1 (4)
C1—C2—C3—C4	1.0 (6)	N1—C8—C9—C10^i^	83.8 (4)
Br1—C2—C3—C4	−179.2 (3)	N1—C8—C9—C10	−157.0 (4)
C2—C3—C4—C5	−1.7 (6)	N1—C8—C9—C8^i^	−35.6 (2)

Symmetry code: (i) −*x*+1, *y*, −*z*+1/2.

## References

[bb1] Allen, F. H., Kennard, O., Watson, D. G., Brammer, L., Orpen, A. G. & Taylor, R. (1987). *J. Chem. Soc. Perkin Trans. 2*, pp. S1–19.

[bb2] Blower, P. J. (1998). *Transition Met. Chem.*, **23**, 109–112.

[bb3] Bruker (2005). *APEX2*, *SAINT* and *SADABS* Bruker AXS Inc., Madison, Wisconsin, USA.

[bb4] Granovski, A. D., Nivorozhkin, A. L. & Minkin, V. I. (1993). *Coord. Chem. Rev.* **126**, 1–69.

[bb7] Kargar, H., Fun, H.-K. & Kia, R. (2008). *Acta Cryst.* E**64**, m1541–m1542. 10.1107/S1600536808036635 PMC2959884 21581155

[bb5] Sheldrick, G. M. (2008). *Acta Cryst.* A**64**, 112–122. 10.1107/S0108767307043930 18156677

[bb6] Spek, A. L. (2009). *Acta Cryst.* D**65**, 148–155. 10.1107/S090744490804362X PMC2631630 19171970

